# Draft Genome Sequence and Gene Annotation of the Entomopathogenic Fungus *Verticillium hemipterigenum*

**DOI:** 10.1128/genomeA.01439-14

**Published:** 2015-01-22

**Authors:** Fabian Horn, Andreas Habel, Daniel H. Scharf, Jan Dworschak, Axel A. Brakhage, Reinhard Guthke, Christian Hertweck, Jörg Linde

**Affiliations:** a Systems Biology/Bioinformatics, Leibniz Institute for Natural Product Research and Infection Biology - Hans Knöll Institute, Jena, Germany; b Biomolecular Chemistry, Leibniz Institute for Natural Product Research and Infection Biology - Hans Knöll Institute, Jena, Germany; c Molecular and Applied Microbiology, Leibniz Institute for Natural Product Research and Infection Biology - Hans Knöll Institute, Jena, Germany

## Abstract

*Verticillium hemipterigenum* (anamorph *Torrubiella hemipterigena*) is an entomopathogenic fungus and produces a broad range of secondary metabolites. Here, we present the draft genome sequence of the fungus, including gene structure and functional annotation. Genes were predicted incorporating RNA-Seq data and functionally annotated to provide the basis for further genome studies.

## GENOME ANNOUNCEMENT

The filamentous fungus *Verticillium hemipterigenum* (anamorph *Torrubiella hemipterigena*) belongs to the phylum Ascomycota. *Verticillium* spp. are commonly known as phytopathogenic species (1). V. hemipterigenum, however, is an insect pathogen and mainly infects leafhoppers belonging to the family Cicadellidae (2). V. hemipterigenum strain BCC 1449 produces a number of highly active secondary metabolites like diketopiperazines and enniatins (3–6).

V. hemipterigenum strain BCC 1449 was grown on Czapek-Dox media and mycelium was harvested after 48 h. Three libraries (paired end [PE], 5 kb mate pair, and 8 kb mate pair) were prepared and sequenced using Illumina HiSeq2000 by LGC Genomics (Berlin). Raw sequence reads were quality trimmed, error corrected (7), digitally normalized (8), and assembled with Allpaths-LG (9). Gaps in assemblies were closed using SOAP GapCloser (10). For transcriptome sequencing, the fungus was grown under three conditions: Czapek-Dox broth, potato dextrose broth, and Sabouraud dextrose broth from BD (Heidelberg). Transcriptome sequencing was performed at LGC Genomics using HiSeq2000 100 bp PE.

Structural and functional gene annotation was performed as described previously. (11, 12). In short, transcriptome assemblies were generated using Cufflinks (13) and Trinity (14) and mapped back to the reference genome using PASA (15). Parameter sets for *ab initio* gene prediction (Augustus [16] and SNAP [17]) were trained using gene models that were predicted by TransDecoder (15) from aligned transcripts. GeneMark-ES (18) was used without training. Transcriptome data were incorporated into Augustus, FGENESH (19), and PASA. In order to create protein alignments, protein sequences from V. dahliae (BROAD) and V. alfalfae (BROAD) were mapped. EVidenceModeler (20) was used to combine gene predictions, while PASA (15) was used to predict untranslated regions. Gene functional annotation was performed using Blast2GO (21) and InterproScan (22). SMURF (23) was used to predict secondary metabolite gene clusters. Gene names and functional descriptions were obtained by blasting against the fungal UniProt Knowledgebase (24).

The genome assembly is based on sequencing 7.0 Gbp, which represents an estimated 240-fold genome coverage. The assembly consists of 26 scaffolds with a total size of 28.5 Mbp (*N*_50_ = 6,006 kbp). Using CEGMA (25), we identified 478 core proteins within the genome. During genome annotation, we utilized RNA-seq data amounting to 21.0 Gbp, which represents an estimated 740-fold genome coverage. Our gene structural annotation pipeline predicted 10,773 genes. Functional annotation resulted in gene ontology categories for 5,845 genes, which have been made available for enrichment analysis with FungiFun2 (26). Additionally, we predicted InterProDomains for 2,749 genes. Prediction of secondary metabolite gene clusters revealed that 404 genes are part of 27 secondary metabolite biosynthesis gene clusters, including 13 PKSs and 16 NRPSs and 4 hybrid PKS-NRPS gene clusters.

### Nucleotide sequence accession numbers.

This genome project was uploaded to DDBJ/ENA/GenBank and is available under the accession numbers CDHN01000001 to CDHN01000026. This paper describes the first version of the genome. Genome data and additional information are also available at the HKI Genome Resource (http://www.genome-resource.de).
